# Quantification of acute myocardial injury in STEMI patients post revascularization at 3Tesla. Comparison of T1-mapping, late gadolinium and edema imaging

**DOI:** 10.1186/1532-429X-13-S1-P146

**Published:** 2011-02-02

**Authors:** Dall'Armellina Erica, Stefan K Piechnik, Vanessa M Ferreira, Theodoros D Karamitsos, Jane M Francis, Matthew D Robson, Robin P Choudhury, Stefan Neubauer

**Affiliations:** 1 OCMR, University of Oxford, Oxford, UK

## Objective

To quantify myocardial injury in patients with acute ST-elevation myocardial infarcts (STEMI) by T1-mapping using Shortened Modified Look-Locker Inversion Recovery (ShMOLLI).

## Background

Acute CMR imaging using late gadolinium (LGE) and T2-weighted imaging (T2W) is used to assess salvageable myocardium in STEMI patients post percutaneous coronary intervention (PCI) . However, current methods of assessing acute myocardial injury are being debated, and a more robust method would be desirable. T1-mapping quantitatively characterizes the myocardium without the need for comparing signal intensities against reference regions (ROI).

## Methods

Seventeen STEMI patients (age 55±8 years) underwent CMR imaging at 3T (TRIO, SIEMENS) 12-48 hours post PCI. Forty-seven matching short axis slices were obtained for cine-SSFP, T2prep-SSFP, LGE, and T1-mapping (ShMOLLI). Segmentation was based on the AHA 17-segment model; volumetric fractions of injured myocardium per segment on LGE and T2W images were calculated using a signal intensity threshold 2SD above the mean intensity of a ROI in remote myocardium. Microvascular obstruction (MVO) identified on LGE images was manually segmented. For quantitative T1-mapping analyses, a threshold value of 1272ms (10% above control T1s of 1156±67 at 3T) was used. Wall motion was scored as normal, hypokinetic and akinetic. T1 values were calculated for segments with varying %LGE lesion and/or wall motion abnormalities (WMA) and for areas of MVO.

## Results

**Figure 1:**
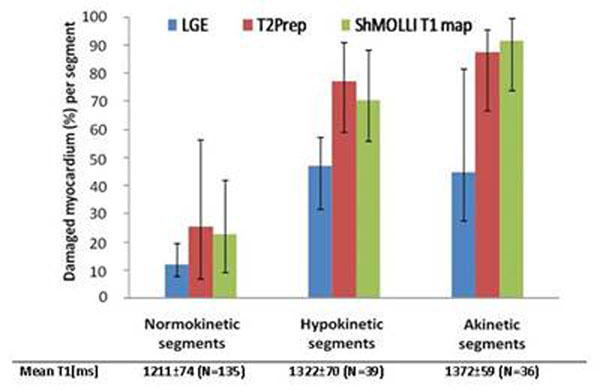
Damaged fraction of LGE, T2p and T2 (median, 25-75% percentiles) per segment grouped by wall motion abnormality (number of segments given in brackets).

**Figure 2:**
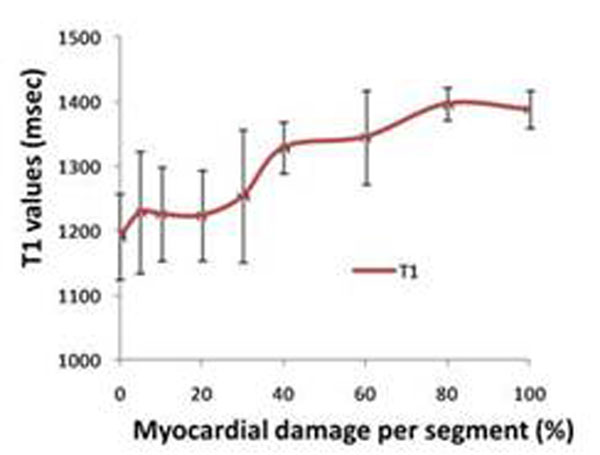
Segments with increasing damaged fraction assessed by LGE show longer T1-values

**Figure 3:**
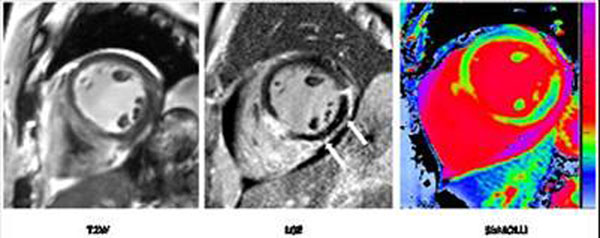
Example of case of inferior myocardial infarction completed by MOV (arrows on LGE image). A short axis slice acquired using three different modalities is displayed.

All patients had positive findings on cine, LGE, T2W and T1-mapping. The regions of myocardial injury assessed by the four modalities co-localized visually. Out of the 282 segments analysed, 39 were rejected for artifacts and 37 were identified as showing MVO and, hence were analysed separately. There was moderate correlation between the segmental damaged fraction assessed by LGE and T2W (r^2^ =0.51) and by LGE and T1-maps (r^2^=0.48). A significant relationship was found between the volume of myocardial injury as assessed by T1-mapping, T2W and LGE, and the severity of WMA (p<0.01). (Fig.1). The T1-values increased with the severity of damage as assessed by LGE (Fig.2) reaching values significantly longer than the T1s in controls for segments with greater than 40% damaged fraction (1356±66ms versus 1233±87ms, p<0.001). T1-values within areas of MVO were abnormal and included both long and short values (1025-1380ms; mean ± SD = 1284±83) while T1s in the myocardium surrounding MVO areas reached values up to 1563ms (Fig. 3).

## Conclusions

ShMOLLI cardiac T1-mapping detects acutely injured myocardium in patients with STEMI and can identify segments with different degrees of damage as assessed by LGE.

